# 3D Object Detection Based on Attention and Multi-Scale Feature Fusion

**DOI:** 10.3390/s22103935

**Published:** 2022-05-23

**Authors:** Minghui Liu, Jinming Ma, Qiuping Zheng, Yuchen Liu, Gang Shi

**Affiliations:** School of Information Science and Engineering, Xinjiang University, Urumqi 830046, China; lmh95810@stu.xju.edu.cn (M.L.); majinming@stu.xju.edu.cn (J.M.); zhengqiuping@stu.xju.edu.cn (Q.Z.); liuyuchen@stu.xju.edu.cn (Y.L.)

**Keywords:** 3D object detection, attention module, voxelization, ConvNeXt module, multi-scale feature fusion

## Abstract

Three-dimensional object detection in the point cloud can provide more accurate object data for autonomous driving. In this paper, we propose a method named MA-MFFC that uses an attention mechanism and a multi-scale feature fusion network with ConvNeXt module to improve the accuracy of object detection. The multi-attention (MA) module contains point-channel attention and voxel attention, which are used in voxelization and 3D backbone. By considering the point-wise and channel-wise, the attention mechanism enhances the information of key points in voxels, suppresses background point clouds in voxelization, and improves the robustness of the network. The voxel attention module is used in the 3D backbone to obtain more robust and discriminative voxel features. The MFFC module contains the multi-scale feature fusion network and the ConvNeXt module; the multi-scale feature fusion network can extract rich feature information and improve the detection accuracy, and the convolutional layer is replaced with the ConvNeXt module to enhance the feature extraction capability of the network. The experimental results show that the average accuracy is 64.60% for pedestrians and 80.92% for cyclists on the KITTI dataset, which is 1.33% and 2.1% higher, respectively, compared with the baseline network, enabling more accurate detection and localization of more difficult objects.

## 1. Introduction

Three-dimensional object detection in point clouds has a large number of applications in realistic scenarios, especially in autonomous driving, robotics, and augmented reality. On the one hand, point clouds have reliable geometric structure information and accurate depth, and how to use this information effectively is a key issue. On the other hand, point clouds are usually disordered, sparse, and unevenly distributed, which poses a great challenge for accurate object detection [1]. Currently, the development of deep learning has led to a proliferation of 2D image object detection algorithms, which can also be applied to 3D point cloud object detection when dealing with sparse and unstructured point clouds.

In recent years, many new 3D object detection algorithms have emerged, which can be divided into two main categories: point-based and voxel-based. PointRCNN [2] operates on each point in the point cloud data, extracts the point cloud features through PointNet [3], and then obtains the final detection results through a two-stage detection network. This has the advantage of making good use of the features of the point cloud and has high detection accuracy, but such a method also has some disadvantages: it needs to collect sample points by farthest point sampling (FPS) [4] and then cluster the features of the proximity points, which takes more time, so the detection speed is slow. Voxel-based methods such as VoxelNet [5] and PointPillars [6] work by dividing the point cloud into regular grids, which is more convenient for convolutional neural networks to operate on it, and because voxels aggregate local features in each grid, they are more efficient for feature extraction and faster detection, but encoding the point cloud into voxels leads to a loss of point cloud feature information and a decrease in detection accuracy.

As the detection technology matures, there is a question of whether the voxel detection method can be improved so that it maintains the existing detection speed and can achieve the point-based detection effect. After analyzing the existing voxel-based detection methods, it is found that they encode the point clouds into voxels by averaging the point cloud features within the voxel grid as the features of the voxel grid, which will undoubtedly fuse the features of the background point cloud. The feature information provided by the background point cloud can interfere with the detection performance of the model, so the background point cloud information needs to be suppressed during voxelization and the foreground point cloud information needs to be better represented. Therefore, the attention mechanism [7] is our first choice because the attention mechanism can make the model focus on the important information through training, and the attention mechanism does not impose a large computational overhead. The attention mechanism in the CBAM [8] and SENet [9] inspired us to research and propose point attention, channel attention, and voxel attention in point clouds. After voxelization, the perceptual field of the feature map is increased by a 3D convolutional neural network; finally, it is transformed into a bird’s eye view (BEV) to perform object detection. It can be found that the voxel-based methods generate some parameters of the object, such as classification information, object position, and size, from the 2D BEV feature map, so the information-rich feature map can help us to obtain better detection performance. We know from [10] that the ConvNeXt module is the best feature extractor now, so this paper adds the ConvNeXt module to the MFF module to obtain richer feature information. It is experimentally demonstrated that the addition of this paper’s method to voxel-based methods can improve their performance, especially in the detection accuracy of smaller objects, such as pedestrians, which is significantly improved.

In summary, the key contributions of the proposed method lie in the following:

- The point-wise attention and channel-wise attention mechanisms are integrated into the process of encoding point clouds into voxels, enhancing the foreground point cloud features, suppressing the background point clouds, and obtaining more robust voxel features by combining different levels of features.
- The voxel attention module is added to the 3D convolutional neural network to represent the voxel grid with more discriminative power.
- The ConvNeXt module is proposed for feature extraction in the multi-scale feature fusion network of BEV, named MFFC, which can extract richer information with greater accuracy and robustness.

## 2. Related Work

With the rapid development of computer vision, at first, people worked on detecting 3D objects from multi-view images, and MV3D [11] fused features from radar laser foreground maps, BEVs, and 2D images to extract regions of 3D objects, but this was computationally expensive and this method could not locate the object very accurately due to the loss of depth information.

In recent years, the research trend of 3D object detection has gradually shifted from RGB images to point cloud data, and point-based methods usually use the features of original point clouds. The use of PointNet [3] for 3D object detection on point clouds was first proposed in FPointNet [12]. PointRCNN [2] generates 3D objects directly from the whole point cloud, instead of projecting the point cloud into 2D images and then detecting them. Part-$A^{2}$ [13] extends PointRCNN by fused point-wise part location features and point-wise semantic features. The Hough voting strategy was proposed in the VoteNet [14] to achieve better point cloud data grouping. LiDAR R-CNN [15] designed a two-stage detector based on original point cloud features. These methods use the PointNet module to aggregate the features of the sampled points.

Voxel-based approaches typically discretize point clouds into equally spaced grids and then perform object detection using 3D convolutional neural networks. VoxelNet [5] was the first to encode point clouds as voxel representations and used PointNet to transform all points within each voxel into a single feature representation. However, there are many voxels in the space that do not contain point clouds, such that the voxels in the whole space are sparse, and feature extraction using traditional convolutional neural networks is inefficient. To reduce the computational cost, SECOND [16] introduces a 3D sparse convolution operation to achieve efficient 3D convolutional processing. PointPillars [6] divide the points into pillars, a pseudo-BEV 2D image is formed after feature extraction of the point cloud within each pillar, and then target extraction is performed using the 2D image target detection method. PV-RCNN [17] extends the SECOND by adding key point cloud branches to retain the structural information of the 3D point cloud and integrates multi-scale voxel features into the key points, generates region suggestions in the BEV image, and further extracts feature from the key points for each 3D region through a region of interest (ROI) pooling operation for anchor refinement. Since the interaction of point cloud features and voxel features in PV-RCNN takes a long time, a detector using only 3D voxel features is proposed in the Voxel R-CNN [18] using a voxel ROI pooling operation to extract region features from 3D voxel features only for further refinement of the anchor. Centerpoint [19] proposes an anchor-free method, which treats the detection target as a key point and enables the network to learn the direction of the object better, compared to the anchor-based method. SE-SSD [20] combines soft information from model prediction and hard information from data annotation to infer the complete target shape and optimize model performance. BtC [21] complements the obscured shapes of the target by learning other point cloud data that match the target shape. It solves the problem of missing shapes in point cloud data due to occlusion and truncation.

All voxel-based 3D object detection methods require the use of voxel feature encoding, so this paper proposes the attention module in terms of point-wise and channel-wise to learn a more discriminative and robust feature representation for each voxel grid. The importance of each voxel is further determined by adding voxel attention to the 3D convolution operation. The region suggestions are generated from the BEV images before feeding into the detection head. Therefore, this paper proposes the MFF-ConvNeXt module, which includes the ConvNeXt [10] module, the current state-of-the-art 2D image object detection structure in the 2D backbone, to replace the feature extraction of the original multi-scale feature fusion network by using depth-separable convolution, inverse bottleneck layer, Gaussian error linear unit, or GELU [22], and larger convolution kernel to obtain richer feature information to be fed into the region proposal network (RPN) to generate better region proposals.

## 3. Method

Before introducing the network, a few basic definitions in 3D target detection are introduced. A point cloud in 3D space is defined as $P={\left\{p_{i}=\left[x_{i},y_{i},z_{i},r_{i}\right]\in R\right\}}_{i=1,2,\ldots ,N}$, where $x_{i},y_{i},z_{i}$ represent the coordinates of each point cloud (X,Y,Z), $r_{i}$ represents the reflection intensity, which is an additional feature of the point cloud, and some point cloud data even carry the color of the point. The feature (R,G,B). *N* represents the total number of points in a point cloud file. An object in 3D space can be represented by a 3D bounding box $\left(c_{x},c_{y},c_{z},h,w,l,\theta \right)$, where $c_{x},c_{y},c_{z}$ represent the center coordinates of the object, h,w,l represent the size of the object, i.e., the length, width, and height, and θ represents the deflection angle of the object around the upward coordinate system.

**Figure 1:**
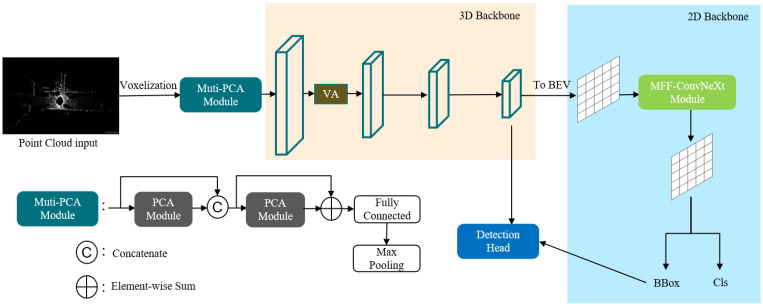
The structure of Voxel R-CNN network with multi-attention module and MFF-ConvNeXt module.

Next, the 3D target detection network based on attention and multi-scale feature fusion will be introduced, and the model in this paper is improved based on Voxel R-CNN, a baseline network with superior performance; the algorithm flow is shown in Figure 1.

### 3.1. Multiple Point-Channel Attention Modules

#### 3.1.1. Point Cloud Voxelization

For the point cloud collection *P* distributed in the 3D space, set its range in the (X,Y,Z) direction denoted as $\left(W_{x},H_{y},D_{z}\right)$, and then divide the point cloud into a grid of voxels of the same size according to the location of the point cloud, each of which has a size of (w,h,d). Therefore, the whole 3D space is equally divided into the size of (W,H,D):

(1) $$W=W_{x}/w$$

(2) $$H=H_{y}/h$$

(3) $$D=D_{z}/d$$

Since the point clouds in 3D space are sparse, the number of point clouds distributed in each voxel grid is also different. Setting *N* and *C* as the maximum number of sampled point clouds in a voxel grid, respectively, the voxel space *V* consisting of *K* voxel grids can be expressed as $V=\left\{V^{1},V^{2},\ldots ,V^{K}\right\}$, where $V^{i}\in R^{N\times C}$ represents the *i*-th voxel grid of the voxel space *V*.

#### 3.1.2. Point-Wise Attention Module

**Figure 2:**
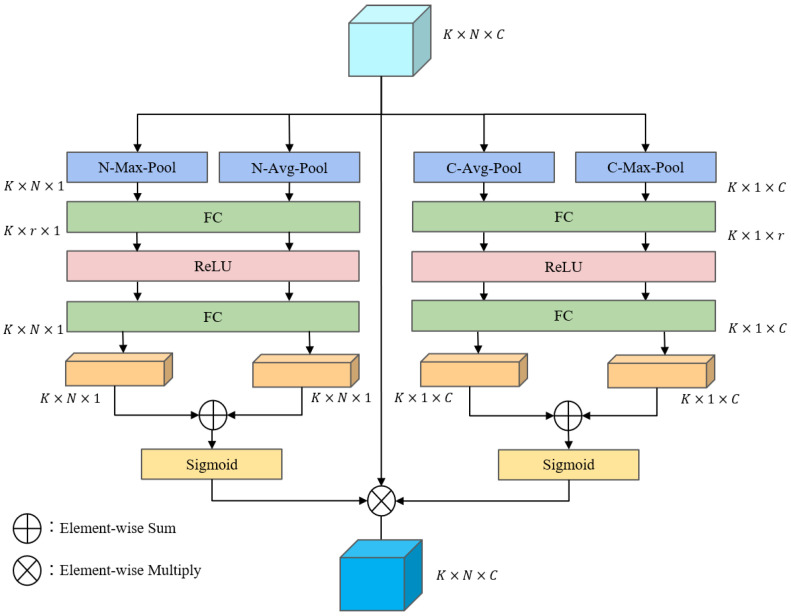
The structure of the point-channel attention module.

For any voxel grid $V^{i}\in R^{N\times C}$ in the space, first perform the maximum pooling operation and average pooling operation to aggregate point features across the channel-wise dimensions. The maximum pooling operation obtains the maximum value of the grid $V^{i}$ in each channel dimension, and the average pooling operation averages the channel features of the grid $V^{i}$ in the channel dimension, resulting in two point-wise responses $A_{1}^{k},A_{2}^{k}\in R^{N\times 1}$, and then uses the shared fully connected layer, ReLU activation function, and fully connected layer to process:

(4) $$M^{i}=\sigma \left(W_{2}\delta \left(W_{1}A_{1}^{k}\right)+W_{2}\delta \left(W_{1}A_{2}^{k}\right)\right)$$

where $W_{1}\in R^{r\times N}$, $W_{2}\in R^{N\times r}$ are the weight parameters of the two fully connected layers, respectively, δ is the ReLU activation function, σ is the sigmoid function, which mainly normalizes the weight features to the range between $\left[0,1\right]$, and $M^{i}\in R^{N\times 1}$ is the point attention weight feature of the voxel grid $V^{i}$, as shown in Figure 2: the left side represents the point attention of the algorithm flow. The point-wise attention module enhances the points with a high contribution and weakens the points with a low contribution.

#### 3.1.3. Channel-Wise Attention Module

The method of channel attention is similar to point attention. As shown in Figure 2, for each voxel grid $V^{i}\in R^{N\times C}$, the maximum pooling operation and the average pooling operation are used to aggregate point features across the point-wise dimensions. The maximum pooling operation obtains the maximum value of the grid $V^{i}$ in each point dimension, and the average pooling operation averages the point features of the grid $V^{i}$ in the point dimension, resulting in two channel-wise responses, $B_{1}^{k},B_{2}^{k}\in R^{1\times C}$, respectively, still using the shared network structure to obtain the channel weight features:

(5) $$L^{i}=\sigma \left(W_{2}^{\prime }\delta \left(W_{1}^{\prime }B_{1}^{k}\right)+W_{2}^{\prime }\delta \left(W_{1}^{\prime }B_{2}^{k}\right)\right)$$

where $W_{1}^{\prime }\in R^{r\times C}$, $W_{2}^{\prime }\in R^{C\times r}$. $L^{i}\in R^{1\times C}$ is the channel attention weight feature of the voxel grid $V^{i}$, which mainly indicates the importance of the feature channels in each voxel grid.

#### 3.1.4. Multiple Attention Module

For the *i*-th voxel grid $V^{i}$, the point attention weight feature and the channel attention weight feature are element-wise multiplied to obtain the attention weight matrix $T^{i}\in R^{N\times C}$:

(6) $$T^{i}=M^{i}\times L^{i}$$

Thus, the features of the voxel grid $V^{i}$ can be obtained from the attention weight matrix and the voxel element-wise dot product, which properly weights the importance of all the points inside a voxel across the point-wise and channel-wise dimensions:

(7) $$O^{i}=T^{i}⨀V^{i}$$

where ⨀ means the element-wise dot product, $T^{i}\in R^{N\times C}$ is the point-channel attention weight of the voxel grid, $V^{i}\in R^{N\times C}$ represents the original point cloud features in the voxel grid, and $O^{i}\in R^{N\times C}$ measures the contribution of points within the voxel in the point-wise dimension and the channel-wise dimension.

The multiple point-channel attention mechanism uses two point-channel attention modules that fuse multilevel features, operates directly on point cloud data in the first module, then connects the inputs and outputs as inputs to the second module, sums the inputs and outputs of the second module to fuse more feature information, obtains a higher dimensional feature representation through a fully connected layer, and finally uses a maximum pooling operation to aggregate all point cloud features within each voxel to obtain the feature representation of the voxel $F=\left\{f^{1},f^{2},\ldots ,f^{K}\right\}$, where $f^{i}\in R^{1\times C}$, as the input to the 3D backbone.

### 3.2. Voxel Attention Module

**Figure 3:**
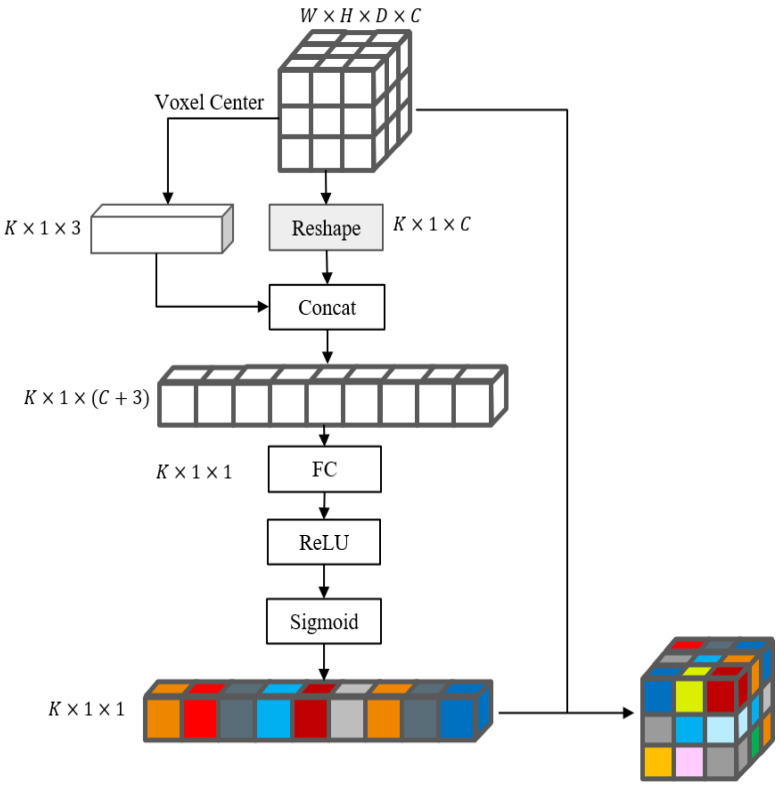
The structure of voxel attention module. The reshape operation permutes the dimension of the tensor from W×H×D×C to K×1×C.

In this paper, the voxel attention module is proposed to determine the importance of each voxel. The structure of the voxel attention module is shown in Figure 3. Set the coordinates of voxel space denoted as $U=\left\{u^{1},u^{2},\ldots ,u^{K}\right\}$, $U^{i}\in R^{1\times 3}$. Firstly, the coordinates of voxels and the features of voxels are connected, the coordinates of voxels can provide accurate position information, and then the voxel weight features $Q\in R^{K\times 1\times 1}$ are obtained by the fully connected layer, ReLU function, sigmoid function, and, finally, the voxel attention weights and voxel features are multiplied by dots to obtain the more robust and discriminative voxel features $F^{V}$:

(8) $$Q=\sigma \left(\delta \left(\left[F,U\right]W_{q}\right)\right)$$

(9) $$F^{V}=Q⨀F$$

where [..,..] means the concat operation, which connects two matrices together in the channel feature dimension, $F\in R^{K\times 1\times C}$ and $U\in R^{K\times 1\times 3}$ denote the voxel features and voxel center coordinates, respectively, so $\left[F,U\right]\in R^{K\times 1\times (C+3)}$. $W_{q}\in R^{K\times (C+3)\times 1}$ is the weight parameter of the fully connected layer. δ is the ReLU activation function, σ is the sigmoid function, which mainly normalizes the weight features to the range between $\left[0,1\right]$. $Q\in R^{K\times 1\times 1}$ means the voxel attention weight. $F^{V}\in R^{K\times 1\times C}$ measures the contribution of voxels.

In the 3D backbone, a voxel attention module is added in the middle of first downsampling of the voxel space, so that the more contributing voxel features can always be filtered.

### 3.3. MFF-ConvNeXt Module

The MFF-ConvNeXt module consists of feature extraction, downsampling, upsampling, and feature fusion. The ConvNeXt module is mainly used for feature extraction, aggregating feature information at multiple scales by 1× and 2× downsampling and the corresponding upsampling.

#### 3.3.1. ConvNeXt Module

**Figure 4:**
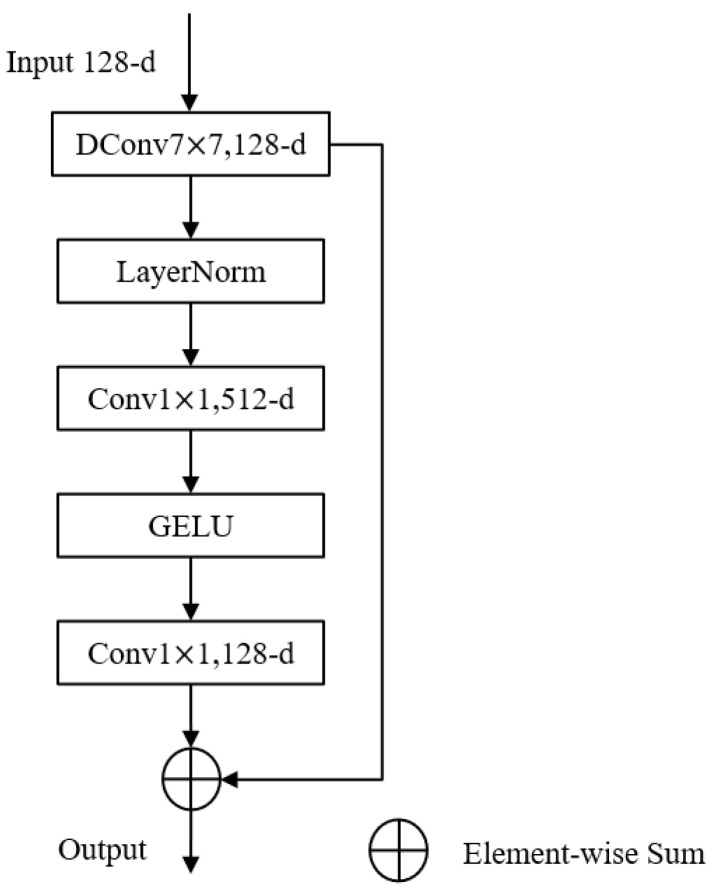
The structure of the ConvNeXt block.

As deep learning continues to emerge in various fields, the original structure of the residual network no longer performs adequately, and ConvNeXt is a model that starts from the original ResNet [23] and improves it by borrowing the design of the swin transformer [24]. Instead of using the common convolutional operations, the ConvNeXt module uses a deeply separable convolution, which consists of a channel-by-channel convolution kernel with a point-by-point convolution. Channel-by-channel convolution has the same number of convolution kernels as the number of channels; one convolution kernel is responsible for one channel, one channel is convolved by only one convolution kernel, and the number of output feature maps is the same as the number of channels in the input layer. The operation of point-by-point convolution is similar to conventional convolution, except that the size of the convolution kernel is 1×1×C, where *C* is the number of channels in the previous layer, and the point-by-point convolution combines the input feature maps pixel by pixel to generate a new feature map. Deeply separable convolution has the advantage of smaller number of parameters and computational effort than conventional convolution. The inverse bottleneck layer structure, which is a structure with a large middle and small ends, was first proposed in MobileNetv2 [25], and such a structure can effectively avoid information loss. The structure of the ConvNeXt block proposed in this paper is shown in Figure 4.

#### 3.3.2. MFF Module

Convolutional neural networks extract features of the target by level-by-level abstraction, and one of the important concepts is the perceptual field. The perceptual field is the range of the region where the pixel points on each layer of the feature map in the convolutional neural network are mapped on the input image. The high-layer network has a large receptive field and a strong feature representation, but the feature map has a low resolution and a weak representation of spatial location features. The bottom layer network has the opposite perceptual field.

**Figure 5:**
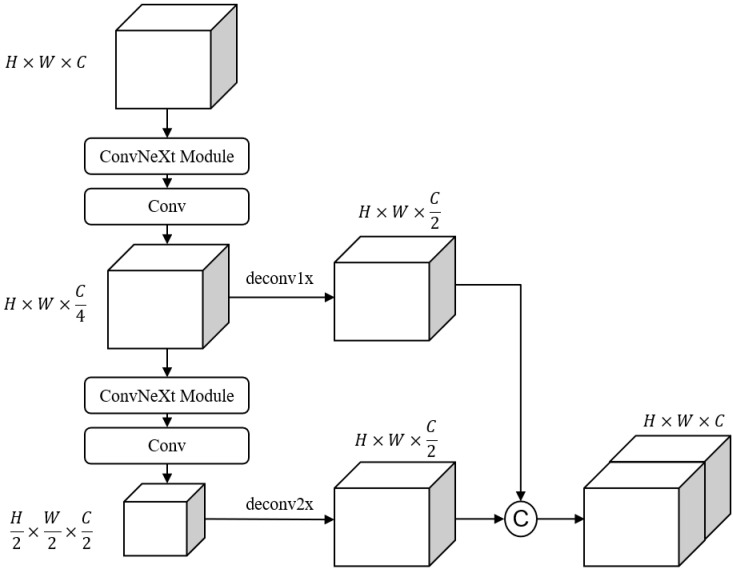
The structure of the MFF-ConvNeXt module.

To solve the problem of small object information loss during downsampling, the usual solution is a feature pyramid network [26], but FPN generates a lot of extra convolutions and multiple detection branches, which increases the computational cost and slower inference speed. This paper borrows the idea of the context enhancement module [27] for increasing the perceptual field and aggregating local and global semantic information from multiple scales to generate more discriminative and richer features. The structure of the multi-scale feature fusion module is shown in Figure 5.

### 3.4. Loss Function

A two-stage object detection network loss function consists of two components, RPN loss, and detection head loss. The RPN loss function can be represented by Equation (10):

(10) $$L_{RPN}=\frac{1}{N_{fg}}\left[\sum_{i}L_{cls}\left(p_{i},c_{i}\right)+I\left(c_{i}>1\right)\sum_{i}L_{reg}\left(b_{i},g_{i}\right)\right]$$

where $N_{fg}$ represents the number of foreground anchor boxes, $p_{i}$ is the output classification score, $b_{i}$ is the output wraparound box regression, $c_{i}$ is the classification label truth value, and $g_{i}$ is the regression target. $I\left(c_{i}>1\right)$ indicates that the regression loss is calculated only for foreground anchor boxes. The focal loss [28] function and smooth L1 loss function are used for classification loss and regression loss, respectively.

The detection head loss is also composed of classification loss and region regression loss, and the target value for classification is first determined based on the *IoU* between the prediction frame and the true frame:

(11) $$l_{IoU}=\left\{\begin{array}{cc} 0 & {IoU}_{i}<\theta_{L}\\ \frac{IoU-\theta_{L}}{\theta_{H}-\theta_{L}} & \theta_{L}\leq IoU<\theta_{H}\\ 1 & IoU>\theta_{H} \end{array}\right.$$

where *IoU* is the intersection ratio of the predicted and real regions, $\theta_{L}$ is the *IoU* threshold for background classification, $\theta_{H}$ is the *IoU* threshold for foreground classification, and the loss function of the detection head can be represented by Equation (12).

(12) $$L_{H}=\frac{1}{N_{s}}\left[\sum_{i}L_{cls}\left(p_{i},l_{IoU}\right)+I\left(IoU>\theta_{\mathrm{reg}}\right)\sum_{i}L_{\mathrm{reg}}\left(b_{i},g_{i}\right)\right]$$

where $N_{s}$ is the number of sampled predictor boxes in the model training phase, $b_{i}$, $g_{i}$ are the predictor box and real box, respectively, $\theta_{\mathrm{reg}}$ is the *IoU* of the predictor box and real box, $I\left(IoU>\theta_{\mathrm{reg}}\right)$ represents that only the predictor box with $IoU>\theta_{\mathrm{reg}}$ contributes to the regional regression loss, the binary cross-entropy loss is used for classification, and the region regression branch also uses smooth L1 loss, as in the RPN.

## 4. Experiments

### 4.1. Datasets

The KITTI dataset [29] contains 7481 training samples and 7518 testing samples in autonomous driving scenes. We follow existing work SECOND [16] to divide the training data into a training set of 3712 frames and a validation set of 3769 frames. When performing experimental studies on the validation set, we use the train set for training. The KITTI dataset contains a total of three classifications: car, cyclist, and pedestrian. Each classification has three difficulty levels: easy, moderate, and hard, depending on factors such as the size of the 3D object, occlusion level, and truncation level. More details about difficulty levels are defined in Table 1.

### 4.2. Implementation Details

#### 4.2.1. Data Augmentation

We randomly sampled some ground truth boxes during the training process and placed them in the samples to increase the number of ground truth boxes in the point cloud and simulate the object in different environments. We rotated the points inside a real 3D bounding box along the *Z*-axis according to a uniform distribution of [−π/4,π/4] to determine the directional variety, and similarly flipped the point cloud inside the 3D box randomly along the *X*-axis or *Y*-axis, scaling the global point cloud randomly in the range of [0.95,1.05].

#### 4.2.2. Voxelization

The raw point clouds are divided into regular voxels before taken as the input of our module. Since the KITTI dataset only provides the annotations of object in FOV, this paper clipped the range of point clouds into [0, 70.4] m for the *X*-axis, [−40, 40] m for the *Y*-axis, and [−3, 1] m for the *Z*-axis. The input voxel size was set as (0.05 m, 0.05 m, 0.1 m), so the voxel size in 3D space was 1600×1408×40. The maximum number of non-empty voxels is set to 40,000, each voxel samples at most 5 points, each point takes the feature (x,y,z,r) as the original input, and for the voxels containing less than 5 points, we pad them with zeros. In the process of voxel feature encoding, the feature size obtained in the first point-channel attention module is 5×8, which is used as the input of the second point-channel attention module. After the output of the second module, the feature of each voxel is 1×4 after passing through the fully connected layer and the maximum pooling layer, and the voxel feature of the whole space is 40,000×4.

#### 4.2.3. Network Architecture

The architecture of 3D backbone follows the design in SECOND. There are four stages in the 3D backbone with filter numbers of 16, 32, 48, and 64, respectively. The structure of the 3D backbone follows the design of [16]. The 3D backbone has four stages with the number of filters as 16, 32, 48, and 64. In the first stage, the voxel attention module is used and the attention weight parameter size is 40,000×1. There are two branches in the 2D backbone, downsampling and upsampling. The first downsampling maintains the same resolution as the input along the *X*-axis and *Y*-axis, and the second downsampling obtains half the resolution of the first, with feature sizes of (64,128) for both downsampling, and each downsampling contains a ConvNeXt module consisting of three ConvNeXt blocks. The upsampling all restores the feature map size to the same resolution as the 2D backbone input and finally connects them to obtain an output of (200×176×256).

#### 4.2.4. Training

The model is trained using the ADAM [30] optimizer with a weight decay parameter of 0.01, and the learning rate is adjusted using a one-cycle [31] strategy with an initial learning rate of 0.00025 and a maximum learning rate of 0.0025. Our model is trained for about 100 epochs with the batch size of 4, and all of our experiments are evaluated on a single GTX 3080 Ti GPU. The threshold $\theta_{H}$ for the foreground IoU is set to 0.75, the threshold $\theta_{L}$ for the background IoU is set to 0.25, and the box regression IoU threshold $\theta_{\mathrm{reg}}$ is set as 0.55. In the detect head, 128 ROIs are randomly sampled, half of which satisfy the condition $IoU>\theta_{\mathrm{reg}}$, contributing to the regional regression loss.

### 4.3. Evaluation on KITTI Dataset

The proposed method was evaluated on the KITTI dataset following the common protocol to report the average precision (AP) of class car with the 0.7 IoU threshold, and the AP of classes cyclist and pedestrian with the 0.5 IoU threshold. The proposed method was tested on the validation set for comparison and analysis. The performance on the validation set is calculated with the AP setting of recall 40 positions.

As shown in Table 2, the proposed method is compared with the state-of-the-art LiDAR-based 3D object detectors on cars, pedestrians, and cyclists using the AP under 40 recall thresholds. We reference the R40 APs of VoxelNet, TANet [32], and PV-RCNN to their papers, the R40 APs of SECOND, PointRCNN, Part-$A^{2}$ [13], PointPillars, and Voxel R-CNN to the results of the officially released code. It can be found that the method in this paper has a 3D mean average precision of 1.17% higher than the current state-of-the-art Voxel R-CNN method, although it is a little lower than the Voxel R-CNN method on the easy and moderate car objects, but 0.17% higher on the hard car objects, outperforming Voxel R-CNN models by 1.85%, 1.21%, and 1.52% 3D R40 AP on the easy, moderate, and hard cyclist objects, respectively, and outperforming the Voxel R-CNN model by 2.12%, 1.86%, and 2.30% 3D R40 AP on the easy, moderate, and hard pedestrian objects, respectively. Our proposed model outperforms TANet by 0.53%, 0.39%, and 0.41% 3D R40 AP on the easy, moderate, and hard pedestrian objects, respectively.

**Figure 6:**
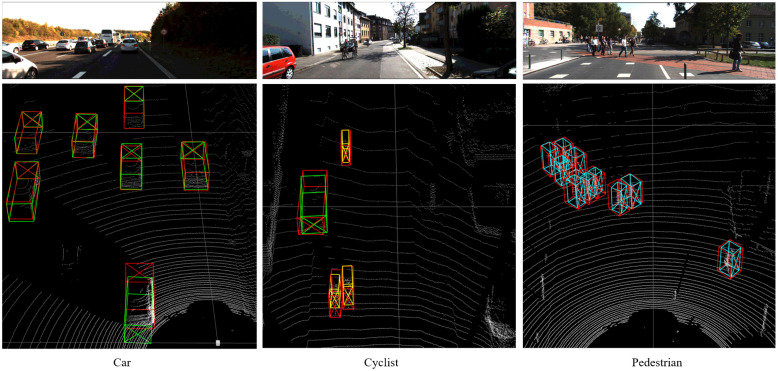
Results of 3D detection on the KITTI validation set. The method proposed in this paper can accurately detect the object in the point cloud. The red box represents the ground truth box of the object, the green box represents the detection result of the car, and the blue and yellow represent the detection result of the pedestrian and the cyclist, respectively.

Figure 6 shows the results of the method proposed in this paper for 3D object detection. The top of the image shows the 2D object in FOV, and the bottom shows the 3D object detection results. In the point cloud, the red box represents the ground truth box of the object, the green box represents the detection result of the car, and the blue and yellow represent the detection result of the pedestrian and the cyclist, respectively. Overall, these evaluation and detection results show that the proposed method can produce accurate results.

### 4.4. Analysis of the Detection Results

**Figure 7:**
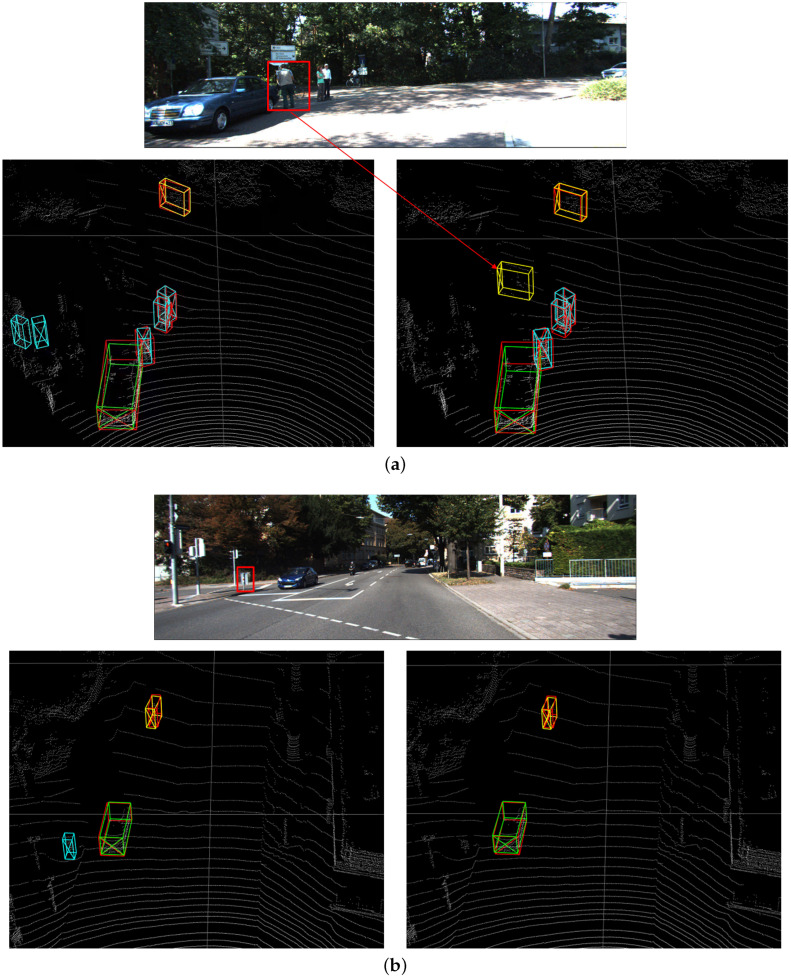
Visualization of 3D object detection result produced by Voxel R-CNN and the method proposed by this paper. The red box represents the ground truth box of the object, the green box represents the detection result of the car, and the blue and yellow represent the detection result of the pedestrian and the cyclist. (**a**) Detection results in obscured scenes. (**b**) Detection results in a simple traffic scenario.

Figure 7 shows the visualization of 3D object detection result produced by Voxel R-CNN and the method proposed by this paper. Each image consists of the FOV at the top, the Voxel R-CNN detection result at the bottom left, and the detection result of this paper at the bottom right.

In Figure 7a, it can be seen that the Voxel R-CNN method identifies the signage and trees in the background as pedestrians, and the proposed method in this paper does not have a false positive. There is also a noticeable lack of labels: some heavily occluded cyclists are not labeled at all, although the network did successfully detect these cyclists.

Figure 7b shows the detection results in a simple traffic scenario. Here, there are some false positive pedestrian detection results. These false positives are present in unreasonable locations in the image. This difficulty can be attributed to the fact that the instance density of pedestrians is usually higher compared to that of cars, which results in pedestrians being more easily confused with other points and noise. In addition, the relatively small volumes of pedestrians lead to less voxels of them. Therefore, with the addition of the attention module, the voxel features of pedestrians are enhanced and these false positives disappear. Compared with the Voxel R-CNN model, the prediction box of cars fits the real box better.

### 4.5. Ablation Studies

Table 3 details how each proposed module influences the accuracy and efficiency of the proposed method. The results are evaluated with mAP of all level for three classes. The baseline is the Voxel R-CNN network. With only point-channel attention (PCA) and only voxel attention (VA), the 3D mAP performance is boosted to 76.44% and 76.37%, respectively. The MFF-ConvNeXt module yields a pedestrians 3D mAP of 63.53%, outperforming the baseline model by 1.03%. When combining PCA and VA, it yields a pedestrians 3D mAP of 63.43%, outperforming the baseline model by 0.93%. Based on the attention module, the MFF-ConvNeXt module was able to obtain rich features, giving significant improvements to the baseline, achieving the pedestrians 3D mAP with about 64.40%, and outperforming the baseline model by 1.9%.

To demonstrate the generality of the attention module proposed in this paper on voxel-based 3D object detection networks, as well as its effectiveness in improving the detection performance of the networks, in this paper, the attention module is added to the two networks, PV-RCNN and Voxel R-CNN, for training, and the evaluation results are shown in Table 4. It can be seen that the mAP of the network is improved after adding the attention module, and the accuracies of cyclist and pedestrian are especially improved significantly.

Finally, Table 5 shows the interfence time between Voxel R-CNN and our proposed method. It can be seen that our proposed method improves by 1.17% on 3D mAP and the inference time increases by only 0.01 s.

## 5. Conclusions

The approach based on attention mechanism and multi-scale feature fusion proposed in this paper for 3D point cloud object detection can effectively improve the detection accuracy, especially for smaller objects such as pedestrians and cyclists. Among them, the method of encoding point clouds into voxels using the point-channel attention module and the voxel attention module can be incorporated into any voxel-based point cloud object detection method, which can effectively facilitate the research of point cloud object detection and other point cloud tasks. The multi-scale feature fusion network introducing the ConvNeXt module can aggregate richer feature information in the 2D backbone, allowing the RPN to produce better box suggestions. The superiority of the network in terms of detection accuracy can be seen through comparative tests. For the two-stage network proposed in this paper, although the accuracy is high, the inference time can be slow. In the future, the network structure can be optimized to improve speeding up the network inference time without reducing the detection accuracy.

## Figures and Tables

**Table 1:** The difficulty level provided by the KITTI dataset.

Level	Min. Bounding Box Height	Max. Occlusion Level	Max. Truncation
Easy	40 Px	Fully visible	15%
Moderate	25 Px	Partly occluded	30%
Hard	25 Px	Difficult to see	50%

**Table 2:** 3D object detection performance: average precision (AP) (in %) and mean average precision (mAP) (in %) for 3D object boxes in the KITTI validation set.

Method	Car 3D			Cyclists 3D			Pedestrians 3D			3D mAP
	Easy	Moderate	Hard	Easy	Moderate	Hard	Easy	Moderate	Hard	
VoxelNet	81.97	65.46	62.85	67.17	47.65	45.11	57.86	53.42	48.87	58.93
PointPillars	87.50	77.01	74.77	83.65	63.40	59.71	66.73	61.06	56.50	70.03
SECOND	89.05	79.94	77.09	82.96	61.43	59.15	55.94	51.14	46.17	66.99
PointRCNN	88.26	77.73	76.67	82.76	62.83	59.62	65.62	58.57	51.48	69.28
Part-$A^{2}$	89.47	79.47	78.54	88.31	70.14	66.93	66.89	59.68	54.62	72.67
PV-RCNN	92.10	84.36	82.48	88.88	71.95	66.78	64.26	56.67	51.91	73.26
TANet	88.21	77.85	75.62	85.98	64.95	60.40	70.80	63.45	58.22	71.72
Voxel R-CNN	92.64	85.10	82.84	92.93	75.03	70.81	69.21	61.98	56.33	76.31
BtC	**93.15**	**86.28**	**83.86**	91.45	74.70	70.08	69.39	61.19	55.86	76.21
Ours	92.60	84.98	83.21	**94.78**	**75.72**	**72.28**	**71.33**	**63.84**	**58.63**	**77.48**

**Table 3:** Performance of the proposed method with different configurations on the KITTI validation set. The results are evaluated with the mAP calculated by 40 recall positions for all classes.

Method	Cars 3D	Cyclists 3D	Pedestrians 3D	3D mAP
Baseline	86.86	79.59	62.50	76.31
PCA	86.87	79.63	62.82	76.44
VA	86.87	79.60	62.66	76.37
MFF-ConvNeXt	86.86	79.75	63.53	76.71
PCA + VA	86.90	79.81	63.43	76.62
PCA + MFF-ConvNeXt	86.92	80.43	64.15	77.16
Ours	86.93	80.92	64.40	77.48

**Table 4:** A comparison of 3D object detection results (mAP) in the KITTI validation set before and after adding the attention module.

Method	Cars 3D	Cyclists 3D	Pedestrians 3D	3D mAP
PV-RCNN	86.31	75.87	57.61	73.26
PV-RCNN + Attention	86.53	78.14	59.32	74.66
Voxel R-CNN	86.86	79.59	62.50	76.31
Voxel R-CNN + Attention	86.90	79.81	63.43	76.62

**Table 5:** Inference time comparison between Voxel R-CNN and our proposed method in the KITTI validation set.

Method	3D mAP	Interfence Time
Voxel R-CNN	76.31	0.13 s
Ours	77.48	0.14 s

## Data Availability

Not applicable.

## References

[1] Guo Y., Wang H., Hu Q., Liu H., Liu L., Bennamoun M. (2020). Deep learning for 3D point clouds: A survey. IEEE Trans. Pattern Anal. Mach. Intell..

[2] Shi S., Wang X., Li H. Pointrcnn: 3D object proposal generation and detection from point cloud. Proceedings of the IEEE/CVF Conference on Computer Vision and Pattern Recognition.

[3] Qi C.R., Su H., Mo K., Guibas L.J. Pointnet: Deep learning on point sets for 3D classification and segmentation. Proceedings of the IEEE Conference on Computer Vision and Pattern Recognition.

[4] Qi C.R., Yi L., Su H., Guibas L.J. Pointnet++: Deep hierarchical feature learning on point sets in a metric space. Proceedings of the 31st Conference on Neural Information Processing Systems (NIPS 2017).

[5] Zhou Y., Tuzel O. Voxelnet: End-to-end learning for point cloud based 3D object detection. Proceedings of the IEEE Conference on Computer Vision and Pattern Recognition.

[6] Lang A.H., Vora S., Caesar H., Zhou L., Yang J., Beijbom O. Pointpillars: Fast encoders for object detection from point clouds. Proceedings of the IEEE/CVF Conference on Computer Vision and Pattern Recognition.

[7] Vaswani A., Shazeer N., Parmar N., Uszkoreit J., Jones L., Gomez A.N., Kaiser Ł., Polosukhin I. Attention is all you need. Proceedings of the 31st Conference on Neural Information Processing Systems (NIPS 2017).

[8] Woo S., Park J., Lee J.Y., Kweon I.S. CBAM: Convolutional block attention module. Proceedings of the European Conference on Computer Vision (ECCV).

[9] Hu J., Shen L., Sun G. Squeeze-and-excitation networks. Proceedings of the IEEE Conference on Computer Vision and Pattern Recognition.

[10] Liu Z., Mao H., Wu C.Y., Feichtenhofer C., Darrell T., Xie S. (2022). A ConvNet for the 2020s. arXiv.

[11] Chen X., Ma H., Wan J., Li B., Xia T. Multi-view 3D object detection network for autonomous driving. Proceedings of the IEEE Conference on Computer Vision and Pattern Recognition.

[12] Qi C.R., Liu W., Wu C., Su H., Guibas L.J. Frustum pointnets for 3D object detection from rgb-d data. Proceedings of the IEEE Conference on Computer Vision and Pattern Recognition.

[13] Shi S., Wang Z., Shi J., Wang X., Li H. (2020). From points to parts: 3D object detection from point cloud with part-aware and part-aggregation network. IEEE Trans. Pattern Anal. Mach. Intell..

[14] Qi C.R., Litany O., He K., Guibas L.J. Deep hough voting for 3D object detection in point clouds. Proceedings of the IEEE/CVF International Conference on Computer Vision.

[15] Li Z., Wang F., Wang N. Lidar r-cnn: An efficient and universal 3D object detector. Proceedings of the IEEE/CVF Conference on Computer Vision and Pattern Recognition.

[16] Yan Y., Mao Y., Li B. (2018). Second: Sparsely embedded convolutional detection. Sensors.

[17] Shi S., Guo C., Jiang L., Wang Z., Shi J., Wang X., Li H. Pv-rcnn: Point-voxel feature set abstraction for 3D object detection. Proceedings of the IEEE/CVF Conference on Computer Vision and Pattern Recognition.

[18] Deng J., Shi S., Li P., Zhou W., Zhang Y., Li H. Voxel R-CNN: Towards High Performance Voxel-based 3D Object Detection. Proceedings of the AAAI Conference on Artificial Intelligence.

[19] Yin T., Zhou X., Krahenbuhl P. Center-based 3D object detection and tracking. Proceedings of the IEEE/CVF Conference on Computer Vision and Pattern Recognition.

[20] Zheng W., Tang W., Jiang L., Fu C.W. SE-SSD: Self-ensembling single-stage object detector from point cloud. Proceedings of the IEEE/CVF Conference on Computer Vision and Pattern Recognition.

[21] Xu Q., Zhong Y., Neumann U. (2021). Behind the Curtain: Learning Occluded Shapes for 3D Object Detection. arXiv.

[22] Hendrycks D., Gimpel K. (2016). Bridging nonlinearities and stochastic regularizers with Gaussian error linear units. arXiv.

[23] He K., Zhang X., Ren S., Sun J. Deep residual learning for image recognition. Proceedings of the IEEE Conference on Computer Vision and Pattern Recognition.

[24] Liu Z., Lin Y., Cao Y., Hu H., Wei Y., Zhang Z., Lin S., Guo B. Swin transformer: Hierarchical vision transformer using shifted windows. Proceedings of the IEEE/CVF International Conference on Computer Vision.

[25] Sandler M., Howard A., Zhu M., Zhmoginov A., Chen L.C. Mobilenetv2: Inverted residuals and linear bottlenecks. Proceedings of the IEEE Conference on Computer Vision and Pattern Recognition.

[26] Lin T.Y., Dollár P., Girshick R., He K., Hariharan B., Belongie S. Feature pyramid networks for object detection. Proceedings of the IEEE Conference on Computer Vision and Pattern Recognition.

[27] Qin Z., Li Z., Zhang Z., Bao Y., Yu G., Peng Y., Sun J. ThunderNet: Towards real-time generic object detection on mobile devices. Proceedings of the IEEE/CVF International Conference on Computer Vision.

[28] Lin T.Y., Goyal P., Girshick R., He K., Dollár P. Focal loss for dense object detection. Proceedings of the IEEE International Conference on Computer Vision.

[29] Geiger A., Lenz P., Urtasun R. Are we ready for autonomous driving? The kitti vision benchmark suite. Proceedings of the 2012 IEEE Conference on Computer Vision and Pattern Recognition.

[30] Loshchilov I., Hutter F. (2017). Fixing weight decay regularization in adam. arXiv.

[31] Smith L.N. Cyclical learning rates for training neural networks. Proceedings of the 2017 IEEE Winter Conference on Applications of Computer Vision (WACV).

[32] Liu Z., Zhao X., Huang T., Hu R., Zhou Y., Bai X. Tanet: Robust 3D object detection from point clouds with triple attention. Proceedings of the AAAI Conference on Artificial Intelligence.

